# Dormancy in Non-Spore-Forming Bacteria: A Review

**DOI:** 10.3390/microorganisms14010074

**Published:** 2025-12-29

**Authors:** Vasili M. Travkin, Nataliya E. Suzina, Inna P. Solyanikova

**Affiliations:** 1 Regional Microbiological Center, Institute of Pharmacy, Chemistry and Biology, Belgorod National Research University, Pobeda Street, 85, Belgorod 308015, Belgorod Region, Russia; travkin-55@mail.ru; 2 Pushchino Scientific Center for Biological Research of the Russian Academy of Sciences, G.K. Skryabin Institute of Biochemistry and Physiology of Microorganisms, Prosp. Nauki 5, Pushchino 142290, Moscow Region, Russia; suzina_nataliya@rambler.ru

**Keywords:** dormancy, non-spore-forming bacteria, vegetative growth

## Abstract

Despite significant breakthroughs in the study of microbial physiology, genetics, ecology, and other disciplines related to these fields of science, there are still quite a few “white spots” in this field. The so-called “resting state” of microbial cells leaves great opportunities for researchers. In this review, we have attempted to outline this problem and define its general nature. We have discussed the physiological processes that lead to the transition of microbial cells into various non-culturable states, as well as related terminological issues. We have also outlined the range of medical concerns and the potential for bio-technology. We have attempted to present the material in a way that reflects the historical development of the problem, and therefore, we have not neglected the bibliographic references from a relatively early period.

## 1. Presence of a Phenomenon

The metabolism of all life forms on Earth depends on the environmental conditions in which these forms exist. Adaptation of the organism to constantly changing external factors is the main condition for survival and is one of the most intriguing problems in biology. It is rightly believed that microorganisms have the greatest degree of adaptability to environmental fluctuations. The adaptability of microorganisms is due to the plasticity of their metabolism, which is controlled by regulatory systems, and environmental conditions that determine the homeostasis of the cell and the population as a whole [1]. Evolutionarily developed and hereditarily fixed mechanisms for the preservation of a species in environmental conditions unfavorable for growth and development include the ability of microorganisms to form dormant forms (DF), which differ in the methods of formation, cell morphology, and dormancy parameters.

## 2. How to Call It? Terminology

Under stressful conditions, a significant number of non-spore-forming bacteria undergo a radical change in their metabolic rate, which is accompanied by a transition to the so-called uncultivated state. Various terms are used to describe this phenomenon: “viable but not culturable (VBNC),” “not culturable (NC),” or “active but not culturable (ABNC)”. The term “Dormancy” is widely used. The problem of the uncultivated state of bacteria has been discussed for quite a long time, for example, in the work of D. B. Roszak and R. R. Colwell [2], and concepts such as “Normal State of Bacteria”, “Viability”, and “Survival” are discussed. The classification proposed by D. Kell et al. back in 1989 [3], which describes various physiological states of the microbial cell with appropriate terminology, should be recognized as exceptionally successful. Thus, the authors in this work differentiate metabolically inactive cells, designating them as dormant, from metabolically active ones. “Active but Not Culturable (ABNC/ANC) is suggested to describe cells which exhibit measurable activity but fail to grow to a detectable level”. However, it should be noted that there is no clearly formulated definition of this state yet in the literature.

In many sources, including the literature devoted to aspects of medical microbiology, the concepts of latency and persistence are often included by authors within the concept of Dormancy. Thus, in a remarkable review, Rittershaus et al. (2013) describe the strategies of adaptation of microorganisms in conditions incompatible with rapid replication using the general concept of “Dormancy” or “Quiescence,” noting that “Dormancy, latency, and persistence are conceptually related terms used to describe the propensity of *Mycobacterium tuberculosis* to arrest its growth in response to host-imposed stress” [4]. Highlighting a number of mechanisms that contribute to “Weather the Storm,” such as “Bust and boom”, “sporulation”, and “quiescence”, the authors, however, point out that “Sporulation is the purest form of microbial dormancy,” apparently implying that other forms of dormancy are not so obvious.

It should be noted that authors often use various terms such as “dormant (resting)”, “anabiotic”, “cryptobiotic”, “sleeping” (“somni cells”), “persistent”, “viable but not culturable”, “active but not culturable”, and “non-growing but metabolically active”, without taking into account the mechanisms of the transition of bacteria to a state of reduced or suspended metabolism, but only record the severity of the external manifestation of the phenomenon. It is possible that different terms reflect the quantitative degrees of suppression of metabolism triggered by the same mechanism [5].

In some cases, “dormancy” is interpreted as a continuum of interrelated states, including stable but non-cultivated (VBNC) states and persistence states [6]. A wide range of arguments for and against the idea that persistent cells are dormant is discussed in the mini-review by Wood, T.K., with coauthors [7].

However, a number of authors emphasize the difference between the terms “Dormancy” and “Persistence”. It was experimentally proven that the absence of replication or low metabolic activity before exposure of pathogen cells to antibiotics only increases the likelihood that the cell is a persister, without guaranteeing persistence [8].

A detailed and quite convincing explanation of the differences between VBNC cells and persistent cells is given in the review [9]. Based on the analysis of a large array of literary data, the authors concluded that VBNC is a deeper stage of dormancy than persistence. Equating a non-replicating state with dormancy and low metabolic activity may be incorrect. While some mycobacterial persisters are indeed in a state of metabolic dormancy, there are also some cells that are physiologically active and have extremely efficient drug efflux, which is responsible for their survival during antibiotic treatment [10,11,12,13]. A more detailed analysis of the phenomenon of persistence, its distinction from other forms, the mechanisms of antibiotic resistance, and even the mathematical definition of this phenomenon can be found in the review [14].

In this regard, it is impossible not to mention a rather intriguing hypothesis of the emergence of persistent cells of “neutral-coupled co-evolution”—population drift in the space of multiple neutrally coupled mutations under conditions of a relative lack of nutritional resources. Some cells acquired the ability to carry out a single cycle of reproduction in several alternative ways, which led to the emergence of persistent cells. Drift ensures phenotypic distability, i.e., the possibility of implementing the reproduction formula: one genotype → two phenotypes [15].

**Figure 1:**
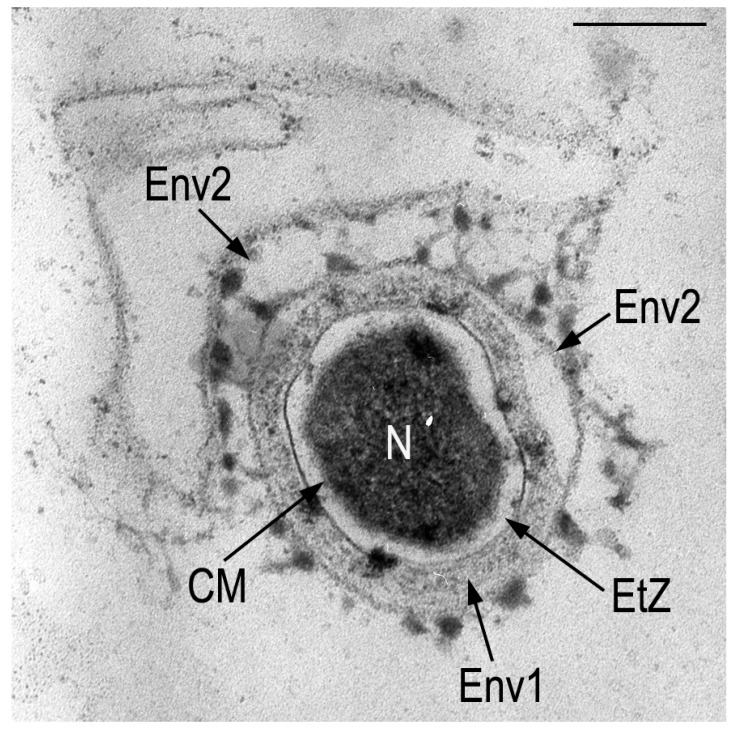
Transmission electron microscopy. An ultra-thin section of a cyst-like cell with multilayered outer envelopes of an unidentified bacterium formed in a zone of extreme temperature fluctuations in the presence of surfactants, found in the formed conglomerate of deposits on the inner surface of a washing machine drainpipe. Designations: CM—cytoplasmic membrane; Env 1—envelope 1; Env 2—envelope 2; EtZ—electron-transparent zone; N—nucleoid. The scale bar—200 nm.

In this review we will not touch upon such strategies of bacterial survival as spore formation, cyst formation, and mycospores, but will try to touch upon the problem of the dormant state in non-spore-forming bacteria, the causes and consequences of this state, denoting this state by the term “dormancy”, fully agreeing with the interpretation of this term by Kaprelyants and coauthors: “a reversible state of low metabolic activity in which cells can survive for long periods without dividing; this often corresponds to a state in which the cells are not “alive” in the sense that they are not capable of forming colonies when seeded on a suitable solid medium, but in which they are not “dead”, since under more favorable conditions they can return to the state of “aliveness”, as defined” [16]. Figure 1 presents the overview of the dormant cell’s structure.

A rather successful, in our opinion, definition of the term dormancy is given by McDonald and colleagues: “a temporary, adaptive, state of reduced metabolic activity within an extended period of arrested growth that enables a microbe to maintain viability under unfavorable environmental conditions.” Understanding this state as an adaptive trait with a multidimensional spectrum of contributing characteristics that combine to enable the capacity for dormancy in a microorganism, it is a fair assumption that different researchers focus on different features associated with the dormant state, but the dormant state can be a consequence of a wide variety of factors and be realized through various mechanisms [17].

## 3. Adaptation or Something Else?

One of the first questions that arose when studying uncultivated (resting) cells was whether this uncultivated state was a state of cell adaptation to stress or a stage preceding cell death.

A fairly comprehensive proof of the adaptive nature of the dormancy state in cells under stressful conditions is given by the work of Sachidanandham and Yew-Hoong Gin [18]. Using *Enterobacter* sp., *Klebsiella pneumoniae* and *Escherichia coli* as examples, the authors showed that the exit of cells from the dormancy state (reversal of dormancy) is accompanied by population asynchrony and the formation of multiple sub-populations within dormant cells. This fact is interpreted as a spontaneous and ordered adaptive response to a stress factor in the external environment. Analysis of the molecular mechanisms underlying the phenomenon of adaptation to osmotic stress shows the formation of a microdiversity in the cell population with different levels (states) of intracellular DNA.

This study expands previously obtained data: in particular, molecular genetic studies on the phenomenon of dormancy under stress conditions (oxidative or nutritional stress) have shown that, in response to stress in prokaryotic cells, there is an over-expression of the Dps protein [19,20,21], which, crystallizing together with DNA, provides large-scale protection of DNA by sequestration [22,23,24]. This mechanism has been proposed as a general strategy for protecting cells under stressful conditions. In general, DNA shielding is accomplished through the formation of two-dimensional Dps-DNA (biocrystalline nucleoid) complexes. In this case, DNA interacts with divalent cations in the protein molecule at certain concentrations.

El-Registan and co-authors studied in detail the structural organization of the biocrystallized nucleoid, described the physicochemical mechanisms of conservation of co-crystallized chromosomal Dps DNA, and explained how nucleoid biocrystallization causes the emergence of a phenotypically heterogeneous population during the germination of dormant cells. One of these phenotypically different variants turns out to be the most adapted to stressful environmental conditions. The process begins with the formation of a compact nucleoid and loops of supercoiled DNA extending from it, which co-crystallize with Dps. Then, a multilayer Dps-Dps structure is formed on the looped Dps DNA. According to the authors, decrystallization of the looped Dps DNA and the subsequent recrystallization lead to Dps binding not only to the first but also to other DNA regions that have an affinity for Dps and, possibly, are partially occupied by other nucleoid-associated proteins. This affects the change in DNA topology and its transcription [25]. These observations conceptually support earlier reports that co-crystallization of stress-induced proteins with DNA is one of the major adaptation mechanisms in bacteria to protect genetic material from environmental assault [26,27,28].

It has also been shown that, when cells are exposed to stress, DNA modifications are induced by “structural phase transitions” that require low energy resources [29]. These studies, together with many other works [30], predicted DNA modification/condensation as an adaptation mechanism in cultured forms of bacteria. At the same time, the nature of cell protection by the Dps protein during stress is bimodal. Zhao et al. showed that, in the case of oxidative stress, the protection mechanism is due to binding Fe^2+^ ions and preventing the Fenton reaction-catalyzed formation of toxic hydroxyl radicals [31]. In this work, the authors presented the stoichiometry of enzymatic oxidation-reduction reactions, where two Fe(II) ions are oxidized per H_2_O_2_ molecule reduced, thus avoiding hydroxyl radical production through Fenton chemistry.

The mechanism of the bimodal nature of the protective effect of the Dps protein under multiple stress impacts on cells was demonstrated in convincing experiments using mutant strains of *E. coli*, defective in DNA-binding or ferroxidase activity [32]. Similar results were obtained by other authors, in particular for the Gram-positive bacterium *Listeria* spp. [33,34,35]. The Dps family of proteins is quite extensive; their functions do not only end with the protection of DNA during the transition to the dormant stage. In particular, their significant role in the infection process is noted [36,37]. The participation of the Dps protein along with other stress proteins in the mechanisms of protection of *Brucella suis* and *Brucella microti* from acid stress without plunging the cells into a dormant state is shown [38].

Quite comprehensive information about this family of proteins can be obtained from a number of works [32,39,40,41,42]. In particular, in the reviews (works) of a number of researchers [23,24,43] along with a description of their role in protecting prokaryotic cells from stress factors, emphasis is placed on the physical, molecular–genetic, and biochemical basis of their functioning.

Trehalose has been shown to play a major role in the formation and maintenance of dormancy in non-sporulating mycobacteria and in exiting this state. Trehalose is known to be an effective protector of biological macromolecules, including DNA, to prevent the denaturation and aggregation of damaged proteins, and to be a cold and heat protector. It has been suggested that there is a relationship between trehalose breakdown and the ATP/glucose level in dormant *Mycobacterium smegmatis*. When the intracellular ATP concentration decreases to a point below a certain critical level (less than 2 mM), trehalose can break down, followed by the formation of free glucose for use in glycolytic reactions and ATP production [44]. Lactate dehydrogenase has also been shown to play a significant role in regulating the dormancy state [45].

Research has shown the considerable complexity and diversity of the biochemical and molecular–genetic mechanisms involved in this process. We provide just a few examples below as an illustration.

It was possible to detect resuscitation-promoting factor (Rpf), a protein secreted by bacteria, which facilitated the exit of several actinobacteria, including *Mycobacterium tuberculosis*, from a state of dormancy [46]. The protein described in the mentioned work was also attributed the properties of a cytokine, as it stimulates the growth of viable cells, and is likely involved in the normal control of cell multiplication [47]. Later, however, this idea was not confirmed for several reasons, including the absence of receptors for this protein on the cell surface. Evaluation of the functional properties of the Rpf protein using Gram-positive strains of *Micrococcus luteus*, *M. smegmatis*, *M. tuberculosis*, *Rhodococcus rhodochrous*, etc., showed that both the introduction of exogenous Rpf and an increase in its expression in the cell led to the reactivation of cells from a nonculturable state [48,49,50,51,52]. The probable mechanism of action of Rpf proteins is associated with their hydrolytic activity. In particular, it is assumed that the key event causing the reactivation of dormant cells from the nonculturable state is the ability of Rpf to hydrolyze and, correspondingly, modify the peptidoglycan in dormant cells, which leads to the synthesis of cell wall components and cell growth. The cleaved peptidoglycans function as signaling molecules for the initiation of growth and resuscitation of VBNC cells [53]. On the other hand, Rpf is able to destroy bacterial aggregates [54].

The family of Rpf-like proteins turned out to be quite extensive, so in the genus *Mycobacterium*, five genes were found whose products resemble Rpf from *M. luteus*. They also stimulate bacterial growth and play a key role in exiting the VBNC state [48]. A comprehensive characterization of Rpf proteins and their probable role in reactivation mechanisms is described in work [55]. Functionally similar YeaZ proteins required for the recovery of the VBNC cells have also been found in Gram-negative bacteria, such as *Salmonella* sp., *Pseudomonas* sp., *E. coli*, *Vibrio parahaemolyticus*, and *Thermotoga maritima* [56,57]. A network of specific proteins, namely YjeE, YeaZ, and YgjD, which are very important for maintaining bacterial viability, has been described [58]. These proteins are involved in the biosynthesis of threonylcarbamoyl adenosine (t6A) [59], which plays an important role in the accuracy of decoding. It has been shown that these proteins form a link between DNA metabolism and cell division.

Reduction of YeaZ expression leads to the formation of cells with highly condensed nucleoids, while the suppression of YjeE and YgjD expression leads to an unusual distribution of DNA at the periphery of cells. YeaZ can hydrolyze peptidoglycan in dormant cells and stimulate the division process in VBNC cells. It is also suggested that YeaZ may post-translationally regulate cellular YgjD pools through proteolytic degradation, which may be related to its effect on VBNC cell repair [60].

In response to a lack of amino acids in the environment and other stress factors, the cell demonstrates the phenomenon of “stringent control” (or stringent response), which is manifested in the formation of the guanosine tetraphosphate molecule (ppGpp) within the cell [61]. The main target of ppGpp action is the promoters of ribosomal RNA genes. Transcription from these promoters is the rate-limiting step in ribosome synthesis, and thus, ppGpp is actively involved in the regulation of cell growth, distributing cellular investments in metabolic proteins and ribosomes, acting as a regulator of the global distribution of bacterial resources. Namely, under conditions of nutrient deficiency, increased ppGpp pools allow the cell to reduce its investments in growth and division, while allocating more proteome resources to metabolic proteins [62]. A number of possible mechanisms for regulating the process of entering and exiting the VBNC state under the control of ppGpp are described, including the possibility of direct control of ribosome activity by ppGpp [63,64,65]. It has been shown that ppGpp mediates the regulation of *rpoS* transcription, encoding the RpoS protein, which is a stationary phase and general stress response regulator [66,67,68,69,70].

Finally, it should be noted that the dormant state can be caused by a number of mechanisms, sometimes quite different. These include:

- Specific DNA repair mechanisms [4,71], involvement of anaplerotic metabolic pathways as components of the stress response, regulated by specific “sensory” proteins and metabolites. Thus, a cascade of proteins has been described that provides an anaplerotic strategy for the transition to the VNBC state for Actinobacteria MI-2665 [6];
- Membrane modifications to maintain fluidity, synthesis of cold-shock, antifreeze, and ice-binding proteins, and cold-adapted proteins in case of cold stress [72,73], as well as a number of others. Obviously, microbial dormancy is not determined by any one mechanism, but, on the contrary, the spectrum of this phenomenon is extremely wide.

Sufficiently comprehensive information on the molecular genetic mechanisms of transition to a state of no growth and the maintenance of viability in it is described in the review by [74]. Various chemical and physical factors that cause the latency state, cell characteristics, and gene expression observed in cells in the VBNC state are well presented in the review [75]. The importance of extracellular adaptive autoregulators for the emergence of the anabiotic state is described in the works of El-Registan et al. Thus, the role of alkylhydroxybenzenes (AHB) in the transition of the microbial cell to the stationary phase and then to the dormant state, their role in the formation of polymorphism of the microbial population, and their role as an autoinhibitor of germination under stressful environmental conditions was shown [76].

## 4. Changes in Population Composition

It was mentioned above that the dormant state in prokaryotic cells is adaptive, including due to the formation of population asynchrony [18]. In experiments with non-spore-forming bacteria, *Enterobacter* sp. strain mcp11b, *Klebsiella pneumoniae* strain mcp11d and *E. coli*, it was shown that the dormant state was accompanied by population asynchrony and the presence of multiple subpopulations in dormant cells.

Using *Arthrobacter oxydans* and *Acinetobacter lwoffii*, exposed to cold stress, a high degree of intrapopulation phenotypic dissociation was shown [77]. Dissociates were characterized by both differences in nutrient needs and high cell resistance to the stress factor. Dormant forms were distinguished by high activity in the formation of phase variants and pronounced polymorphism, which, according to the authors, underlies significant adaptive potential. The same group of researchers showed the high potential of using MALDI mass spectrometry of proteins as a promising technique for the further development of methods of detecting the intrapopulation phenotypic variants and for research on bacterial intrapopulation dissociation of cells in a dormant state [77,78].

**Figure 2:**
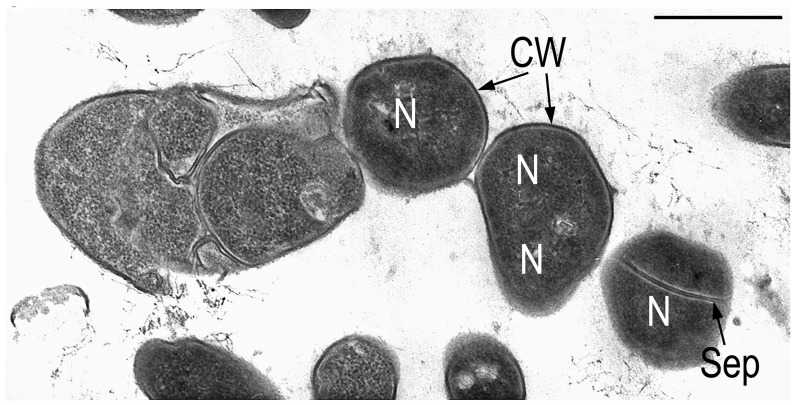
Transmission electron microscopy. Ultrathin section of disintegration of multicellular conglomerate cyst-like dormant cells *Microbacterium foliorum* BN52 into unicellular forms at later stages of germination. Designations: N—nucleoid, CW—cell wall, Sep—septum. Scale bar: 500 nm.

A number of studies, including our own [25,28,76,79], have shown that, at the moment of transition from a state of rest to vegetative growth, the mechanism of formation of large polyploid cells is activated with the simultaneous suppression of cell division. This process is accompanied by functional restructuring of the genome. The subsequent division of polyploid cells leads to the formation of numerous viable small-cell forms, each of which contains one nucleoid (Figure 2). The chromosomes of these cells can differ in the expression of certain features, which leads to phenotypic diversity and the activation of previously unobserved physiological and biochemical properties [79,80].

On the other hand, using the strain *Pseudomonas fluorescens* 26 K as an example, we showed that the use of inoculum obtained after the transfer of germinated dormant forms did not have a strong effect on improving the degradative activity of the strain in relation to a wide range of xenobiotics, although dormant cells retain long-term viability, have increased resistance to adverse effects, and are characterized by a specific ultrastructural organization. We associate the lack of progress in biodegradative activity primarily with the instability of minor intrapopulation variants, which quickly return to the dominant phenotype under laboratory experimental conditions [81].

The study of dormant forms of the actinobacteria *Microbacterium foliorum* BN52 allowed us to draw a number of conclusions about the adaptive role of the dormant state and possible survival strategies of actinobacteria exposed to toxicants.

The first type of strategy is based on the ability of bacteria from communities of contaminated ecotopes, which are (almost) incapable of utilizing toxic compounds as growth substrates, to use available non-toxic organic compounds and to develop during the periods of decreased toxic load, e.g., at increased soil humidity, switching to the state of metabolic rest at elevated toxicant concentrations. At the cellular level, this dormant state is associated with the formation of cyst-like dormant (anabiotic) cells (CLC), which have been described previously for a number of non-spore-forming bacteria [76,82] and which were revealed in the *M. foliorum* strain BN52 [83].

**Figure 3:**
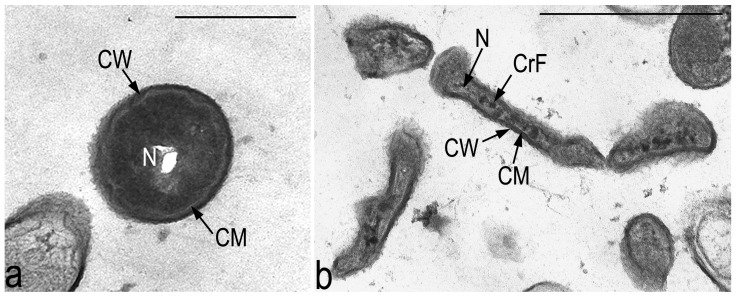
Transmission electron microscopy of ultrathin sections. Two morphotypes of cyst-like resting cells can be formed in a population of bacteria of the genus *Microbacterium*: (**a**) small spherical cells with a compacted nucleoid and very dense cytoplasm and cell wall. (**b**) Filiform elongated cellular formations with an intact cell envelope, sparse cytoplasm, and crystal-like structures in the nucleoid zone. Designations: CW—cell wall; CM—cytoplasmic membrane; N—nucleoid; CrF—crystal-like structures. Scale bar—500 nm.

The second type of strategy is the survival strategy of such non-degrading bacteria, which is supported by pronounced intrapopulational variability, which is characteristic of nonmycelial actinobacteria [78]. This variability is represented by the phenotypic heterogeneity of the populations of both the dormant forms, morphotypes I and II CLC, and the vegetative cells of the S, R, and microcolonial types, which develop upon the germination of the dormant forms (Figure 3). Instability of the vegetative phenotypes and their reversibility provide additional adaptive potential to the studied strain.

Conclusions regarding the third type of strategy were made based on the most interesting priority results of the investigation of the ultrastructural reorganization of dormant CLC on their exit from the dormant state, germination, and the beginning of a new vegetative cycle. Formation of large polynucleoidal cell forms was shown to occur at the initial stages of CLC germination (especially of morphotype I CLC). Transition to vegetative growth probably switches the process of replication of chromosome copies with simultaneous suppression of cell division, which results in the formation of large polyploids. This process may quite probably be accompanied by a functional reorganization of the genomes [83].

Similar results indicating the adaptive nature of the dormant state were also shown for the soil *Arthrobacter agilis* lush13. Under conditions of growth arrest due to nutrient depletion or the inability to use some ecotoxicants as the only carbon sources, a significant part of the population of the studied strain during the experiment adopted a dormant state in the form of conglomerates of small and ultra-small cyst-like cells responsible for long-term survival. Germination of dormant small and ultra-small cells was accompanied by the destruction of conglomerates with the release and further division of small daughter cells [84]. In addition to the above-mentioned strains, population variability is also characteristic of bacilli and pseudomonads [85,86].

## 5. The Prospect of Using the Dormant Stage of Non-Spore-Forming Bacteria

All of the above could not help but prompt researchers to think that the dormant state may be an effective tool for creating microorganisms with specified physiological and biochemical properties. The process of exiting the dormant state of the already mentioned strain *Rhodococcus opacus* 1CP [79] demonstrated the effect of the appearance of phenotypic variants that could grow on a wide range of substrates that were not previously utilized by this strain: 4-chlorophenol, 2,4,6-trichlorophenol, 2,3-, 2,5- and 2,6-dichloro-, 2,3,4- and 2,4,5-trichloro-, pentachlorophenol, and 1,2,4,5-tetrachlorobenzene.

An assessment of the growth parameters of this strain on benzoate and its chlorine-substituted derivatives, as well as a study of the properties of key enzymes of biodegradation of this group of compounds, showed that, to resume growth after leaving the dormant state, the strain can activate various enzyme systems. Thus, the substrate specificity of catechol 1,2-dioxygenase (Cat 1,2-DO) isolated from cells of strain 1CP after dormancy was found to differ significantly from that of Cat 1,2-DO isolated earlier from cells of this strain grown on benzoate. Thus, it can be assumed that the mechanisms of enzyme induction are determined not only by the substrate, but also by the physiological state of the culture [87]. Ivshina and coauthors studied the degradation of drotaverine hydrochloride by the *Rhodococcus ruber* strain IEGM 326. The results of the experiments showed that the use of dormant cells as an inoculum at a non-optimal temperature led to more efficient degradation of the xenobiotic [88]. 

In the natural environment, the degradation of xenobiotics slows down if the degrader cells enter the VBNC state under the influence of external factors—temperature, carbon starvation, etc. In this case, the introduction of a resuscitation-promoting factor (Rpf) may be a promising biotechnological technique. In soil microcosms inoculated with the *R. biphenylivorans* strain TG9T, exogenous Rpf resuscitated TG9T cells that had previously entered the VBNC state, and this led to a significant increase in the degradation of polychlorinated biphenyl. Exogenous Rpf resuscitated VBNC TG9T cells by stimulating the endogenous expression of *rpf* gene orthologs [89,90].

An indirect but nevertheless logical argument for dormancy leading to a change in the transcriptional response to a xenobiotic comes from data on the degradation of sulfanilic acid by the bacterium *Novosphingobium resinovorum* SA1. The authors report that a comparison of transcript profiles showed that the main effect of sulfanilic acid on the cell transcriptome was a starvation-like effect [91]. It should be noted that this study refers to starved, but not dormant, cells, which is nevertheless also of interest in terms of the regulation of biodegradation pathways.

Carbon starvation significantly increased the level of regulation of the arsenite oxidase gene of bacterial cultures isolated from tannery effluent [92].

This suggests the importance of the dormant state for the development of the adaptive potential of populations and a new approach to the selection of microorganisms with the required biotechnological properties. Listing all the fundamental biochemical, physiological, and genetic changes that occur in cells during the transition to a dormant state, Moens, E., and co-authors [93] propose calling dormant cells non-growing metabolically active cells or NGMA cells. Considering the resistance of such cells to the often unfavorable cultivation conditions in industrial biotechnology, their altered metabolic properties, the authors substantiate the prospects of their use in biotechnological processes, including for the production of difficult-to-synthesize secondary metabolites.

## 6. Biomedical Aspects

The influence of the dormant state on the physiological and biochemical properties of the microbial population cannot help but affect a very serious problem, namely, the biomedical aspects of this phenomenon. Using the example of the non-spore-forming bacteria *Staphylococcus aureus* and *Corynebacterium pseudodiphtheriticum*, it was shown that the exit from the dormant state of the cells of these cultures was accompanied by phenotypic variability, including the morphological diversity of colonies and, what is very important, the emergence of antibiotic-resistant strains [94]. The latter fact forces the authors of this study to pose the question of whether the population of dormant forms of opportunistic and pathogenic bacteria in environmental objects may act as a source of antibiotic-resistant clones. The same problem—acquired antibiotic resistance—is also addressed by the authors in their earlier studies [86,95]. Using the example of the pathogens of the hospital-acquired infections *Moraxella catarrhalis* and *Kocuria rhizophila*, it was shown that, in a dormant state, the cells retained viability under stressful conditions and could be responsible both for survival in the environment and persistence in the host organism [96].

There is another issue. It has been shown that cells in a dormant state are able to maintain virulence [97,98]. But, since in a dormant state they are not detected by traditional methods, they represent a serious problem for public health [97,99,100]. In addition to other problems caused by pathogenic microflora, the change in the morphology of *Burkholderia cepacia* complex cells from rod-shaped to coccoid in the dormant state makes it possible for cells to “slip through” during filtration sterilization, which poses a real contamination risk in water-based pharmaceutical products [101]. Potentially dangerous microorganisms, due to the dormant state, are able to remain viable for many years [102] and increase tolerance to antibiotics [103,104].

In the dormant state, genes involved in the redox processes of the *Staphylococcus epidermidis* biofilm were more active, and in the same biofilm, genes associated with ribosome activity were upregulated [105]. The authors discuss whether there is a lower virulence potential in dormant cells due to lower metabolism or whether the expression of a certain gene or set of genes can lead to suppression of the host immune response. At the same time, there is evidence that, in some cases, the level of virulence decreases in pathogenic microorganisms in the VBNC state. Thus, subpopulations of *Vibrio parahaemolyticus* RIMD2210633 cells in the VBNC state were unable to recover when introduced into the host organism. From the pool of known virulence proteins, the authors found only eight proteins (4%) [45].

Dormant microbes in the human body play a dramatic role in the development of chronic inflammatory diseases. Using the strategy of systems biology, Douglas B. Kell and Pretorius showed that both external (e.g., trauma) and internal factors (e.g., diet) promote the release of iron, which is necessary for the beginning of replication of dormant cells. Their replication triggers the process of inflammation, which results in a whole range of diseases, such as Alzheimer’s disease, Parkinson’s disease, rheumatoid arthritis, multiple sclerosis, etc. The arguments collected by the authors in favor of this hypothesis (Iron Dysregulation and Dormant Microbes (IDDM)) hypothesis of chronic inflammatory diseases are more than convincing [106].

## 7. Preserve or Prevent the Survival of Non-Spore-Forming Bacteria: The Result Is Determined by the Goal

A key aspect emerging from the study of bacterial dormancy is the potential for exploiting this phenomenon for biotechnological purposes. Moreover, depending on the technological area that is affected by microorganisms, one may be concerned with both the preservation of the viability of non-spore-forming bacteria and their destruction. For example, one of the challenges associated with the use of bacterial cell-based biopreparations (e.g., biopreparations for environmental bioremediation) is their shelf life due to the loss of cell activity. Immobilizing bacterial cells in a gel has proven to be an effective approach to preserving bacterial viability. The cell state in the gel, on the one hand, is similar to that of the bacteria in biofilms, ensuring the preservation of the viability and activity of microbial populations. On the other hand, it has been shown that, if the gel contains humic compounds, they stimulate the formation of stress-resistant, persistent cells [107,108,109,110,111].

The opposite situation arises when it is necessary, for example, to get rid of unwanted microflora to prolong the shelf life of food products. Thus, non-thermal processing of food products is used as an alternative to thermal processing and helps maintain the high quality of products. However, this approach can only be used to remove non-spore-forming bacteria, as spore-forming bacteria are resistant to this method of processing. A number of advantages of non-thermal food processing are well covered in the review by Wu, Y. and co-authors, who point out its advantages, such as “lower energy consumption, enhanced environmental sustainability, effective microbial inactivation resulting in extending food shelf life” [112]. However, despite the obvious advantages of this method, a number of problems can be identified that are associated with the survival of bacteria that can enter the NCBV state [113,114]. Thus, the authors emphasize that the impact of one stress on a microorganism can lead to an increase in its resistance to other stresses, since, as a result of the adaptive response, resistance to the effects of damaging and other factors increases. Moreover, insufficient exposure to a stressor can lead to the restoration of the physiologically active state of the cell, which will lead to the re-growth of the microorganism [112,113,114].

## 8. For the Conclusions

Despite the fact that bacterial dormancy has been studied for over 30 years, this area of research remains unfinished and continues to generate considerable debate among researchers. It is a fact that VBNC is a way to cope with stress. Nevertheless, research into the physiological, biochemical, and genetic mechanisms of entering, maintaining, and exiting the VBNC state represents a vast field of study for scientists. In the VBNC state, bacteria maintain cellular structure, respiration, and continuous gene expression, i.e., the proton motive force (PMF) functions. However, upon exiting the VBNC state, the phenotype of the new population can significantly change, revealing both positive and negative properties, necessitating a more careful examination of this phenomenon. By defining bacterial dormancy as a multidimensional feature space, McDonald and co-authors propose a pluralistic concept and clearly justify the need to employ multi-omics tools to unravel the phenomenon of dormancy [17]. Undoubtedly, alongside purely utilitarian interests (medical aspects, biotechnological aspects, including the food industry, environmental protection, agriculture, etc.), the phenomenon of dormancy in bacteria remains a profound philosophical problem, the study of which contributes to a deeper understanding of the phenomenon of life strategies on the planet.

## Data Availability

The data presented in this study are available on request from the corresponding author.

## Abbreviations

ABNC—active but not culturable; DF—dormant forms; IDDM—Iron Dysregulation and Dormant Microbes; CLC—cyst-like cell; NC—not culturable; Rpf—resuscitation-promoting factor; VBNC—viable but not culturable.
